# Establishing a journal club to promote professional development within a trials unit

**DOI:** 10.1186/1745-6215-16-S2-P202

**Published:** 2015-11-16

**Authors:** Fiona Strachan, Chris Tuck, Gina Cranswick, Lorraine Smith

**Affiliations:** 1 University of Edinburgh, Edinburgh, UK

## 

Journal clubs provide an informal way of discussing research and how it applies to practice. We established a journal club within the Edinburgh Clinical Trials Unit to stimulate discussion of research around trial conduct and methodology and how research applied to staff within the unit. Our aims were to:

• Support professional development

• Keep up-to-date with the literature around clinical trial methodology

• Disseminate information on and build up debate about good practice

• Ensure that professional practice is evidence-based

• Learn and practice critical appraisal skills

• Develop ideas for research, descriptions of good practice within the unit that could be presented at meetings and/or submitted for publication

• Provide an enjoyable educational and social occasion

The journal club is led by two trial managers and all staff are invited. Meetings are held monthly with a mix of discussions on published papers and presentations from the team.

We conducted an online survey to get feedback on our first year, and received 21 responses (18 completed and 3 not completed). The feedback was positive and there was general agreement that we had achieved our aims, in particular that journal club was an enjoyable educational and social occasion (100%) and helped disseminate information on and build up debate about good practice (94%).

In summary, journal club has been a positive initiative and has led to more discussion within the group on published research and how this applies to our practice.

